# The role of Tat in HIV latency and reactivation

**DOI:** 10.3389/fimmu.2025.1650385

**Published:** 2025-08-27

**Authors:** David M. Margolis, Edward P. Browne

**Affiliations:** Departments of Medicine, Microbiology and Immunology, UNC HIV Cure Center, University of North Carolina at Chapel Hill, Chapel Hill, NC, United States

**Keywords:** HIV, Tat, latency, persistence, clearance

## Abstract

HIV persists during therapy due the existence of a latently infected reservoir in which viral gene expression is silenced. This reservoir thus represents the primary barrier to a cure for HIV. To eliminate latently infected cells from people with HIV (PWH) on antiretroviral therapy (ART), small molecules that reverse HIV latency (Latency reversing agents – LRAs) have been previously developed and tested, but these lack specificity for HIV and are typically inefficient at promoting broad reservoir reactivation. As such, more potent and selective tools for latency reversal are needed. Recently, delivery of mRNA encoding the viral protein Tat, which promotes transcriptional elongation, has attracted interest as a possible HIV-specific approach to inducing latency reversal. This review will cover the evidence that Tat plays a key role in both establishment of HIV latency and latency reversal, as well as recent developments in which Tat mRNA delivery has been used to enhance latency reversal approaches. Delivery of Tat to infected cells represents a promising avenue to bypass the limitations of small molecule LRAs and achieve broad reactivation of the clinical reservoir.

## 1 HIV latency

Although antiretroviral therapy (ART) potently suppresses HIV replication and allows people with HIV (PWH) to lead relatively normal lives, interruption of ART results in rapid viral rebound (1, 2). HIV persists during ART due to the formation of a latently infected reservoir of cells, in which viral gene expression is reduced or absent. This latent reservoir is established throughout untreated infection, although recent evidence suggests that the reservoir is enriched with viruses circulating at time of therapy initiation (3, 4). Over time on ART, the reservoir becomes increasingly composed of defective proviruses that contain large inactivating mutations or G to A hypermutation, likely due to intact proviruses with residual expression being counter-selected by the toxicity of viral proteins to the cell, or by the antigen-specific killing of these cells by the immune system (5). Nevertheless, a reactivation-competent and replication-competent reservoir remains that can sporadically reactivate and reinitiate viremia after treatment interruption, even after decades of ART. As such, curing HIV will require the elimination of this reservoir or its functional containment by the immune system.

The mechanisms by which HIV establishes the reservoir of latently infected cells is an area of active investigation. Studies from *in vitro* models have shown that, following infection, HIV can enter a state of reversible transcriptional quiescence in a subset of infected cells (6–8). This phenomenon can occur following *in vitro* infection of cell lines of non-T cell origin, suggesting that it is not unique to resting CD4 T cells, the primary cellular location of the reservoir in peripheral blood (9). Moreover, latent proviruses can be reactivated and initiate the release of infectious virus particles, either sporadically or after exposure to latency reversing agents (LRAs). The precise molecular causes of HIV latency are not totally clear but, in latently infected cells, expression of viral genes can be blocked at several steps, including transcriptional initiation, elongation, splicing, RNA export and translation (10–12). We, and others, have previous postulated that latency represents not a single state, but a heterogeneous set of states with diverse and overlapping combinations of restrictions present in different subsets of cells (13, 14). Key limiting factors for viral expression are thus likely heterogenous across the reservoir, explaining the inefficient nature of single agent LRAs.

## 2 Latency and the host cell

HIV expression and replication involves the activity of numerous host cell factors, leading to the hypothesis that latency can result from the lack of a positive host factor or from the presence of a repressive host cell factor. HIV proviruses are integrated into the host cell genome and are transcribed by the host cell RNA Polymerase II (RNAPII), and are thus subject to modes of regulation common to cellular genes. The HIV U3 region within the long terminal repeat (LTR) contains promoter and enhancer sequences that include binding sites for cellular transcription factors (TFs) that regulate HIV expression, including NF-κB, SP1 and AP-1, and lack of nuclear availability or activity for these TFs can induce a state of latency (15–17). Furthermore, changes to these TFs binding sites can affect HIV expression and can promote latency (18). Latently infected cells also express a set of host cell proteins that can inhibit HIV expression, either directly or indirectly including NELF, DSIF, CTCF, ETS1 and INTS12 (19–24). Single cell RNA sequencing studies of HIV infected CD4 T cells have also revealed that HIV expression and latency is correlated with several hundred cellular genes, and that silencing occurs preferentially in cells with a quiescent stem cell memory or central memory phenotype (25, 26). Transcription of latent HIV proviruses is also inhibited by enzymes that mediate covalent modifications to provirus associated histones, such as histone deacetylases and histone methyltransferases that, together, promote the formation of heterochromatin across the provirus that restricts transcription, and leads to a “closed” conformation at the viral promoter that prevents TF binding (14, 27–31). Altogether, these data indicate that, although latency is a frequent outcome of infection that can occur in different host cell types and tissue environments, HIV latency is nonetheless influenced by the intracellular environment of the host cell.

## 3 Restriction of HIV transcriptional elongation in latently infected cells

Studies in cell-based models and in cells from people with HIV have indicated that an important block to HIV transcription in latently infected CD4 T cells occurs at the level of transcriptional elongation (12, 32). Following the formation of a transcriptional initiation complex at the viral LTR and promoter clearance, HIV transcription is subjected to pausing at 62 nucleotides, following transcription of the Trans Activation Response element (TAR). This pausing is stochastic in nature but is long-lived (>20 minutes) and limits the overall rate of HIV gene expression by up to 100-fold (32, 33). TAR is a highly structured RNA with a stem-loop-bulge structure that interacts with viral and cellular proteins (34–36). In particular, release of pausing for HIV requires the action of the cellular P-TEFb complex, comprised of Cyclin T1 and CDK9 (37–40). During transcriptional activation, P-TEFb is recruited to the TAR RNA element and forms a ternary complex that phosphorylates serine2 of the C-terminal tail of RNAPII, allowing transcription to continue through the pausing site, as well as phosphorylating the pausing-enhancing factors NELF and DSIF, promoting their release (NELF) or conversion into a positive elongation factor (DSIF) (21, 39–41). In resting CD4 T cells, the level of P-TEFb has been shown to be low and likely limiting for HIV transcription (42). Furthermore, P-TEFb in latently infected cells can be sequestered in complexes such as the 7SK complex (43). Recent data have also indicated that P-TEFb activity can be limited in latently infected cells by sequestration of P-TEFb in super-elongation complexes (SEC) and that small molecule inhibitors of the P-TEFb-SEC interaction can promote latency reversal (37).

## 4 Tat and viral latency

The HIV protein Tat is known to potently enhance HIV transcription (44). The full-length Tat gene encodes a 101 amino acid protein that is expressed early in infection from a multiply spliced transcript, although an 86 amino acid variant of Tat is also found in infected cells (45). Initial HIV expression is Tat-independent and heavily restricted at the level of transcriptional elongation, as described above, but a low level of full-length HIV transcripts is made leading to an initial low burst of Tat protein in infected cells. A key function of this initial pool of Tat is to bind the TAR element and promote recruitment of P-TEFb to paused transcription sites, thereby promoting a feed-forward dynamic that greatly enhances overall HIV expression (46). Lack of sufficient Tat expression during early infection thus can lead to failure to amplify viral gene expression, leading to the establishment of a latent state. Mathematical modeling of the role of Tat in viral transcription in HIV infected cells indicates that a model in which stochastic toggling of the promoter between an on and off state, combined with a feed-forward Tat network, leads to a bifurcation of Tat levels early in infection (47, 48). Thus, the ability of HIV to enter a state of reversible latency may, in part, be a ‘hard-wired’ feature of the virus itself, with the periodic accumulation of Tat above a threshold level playing a key role.

**Figure 1:**
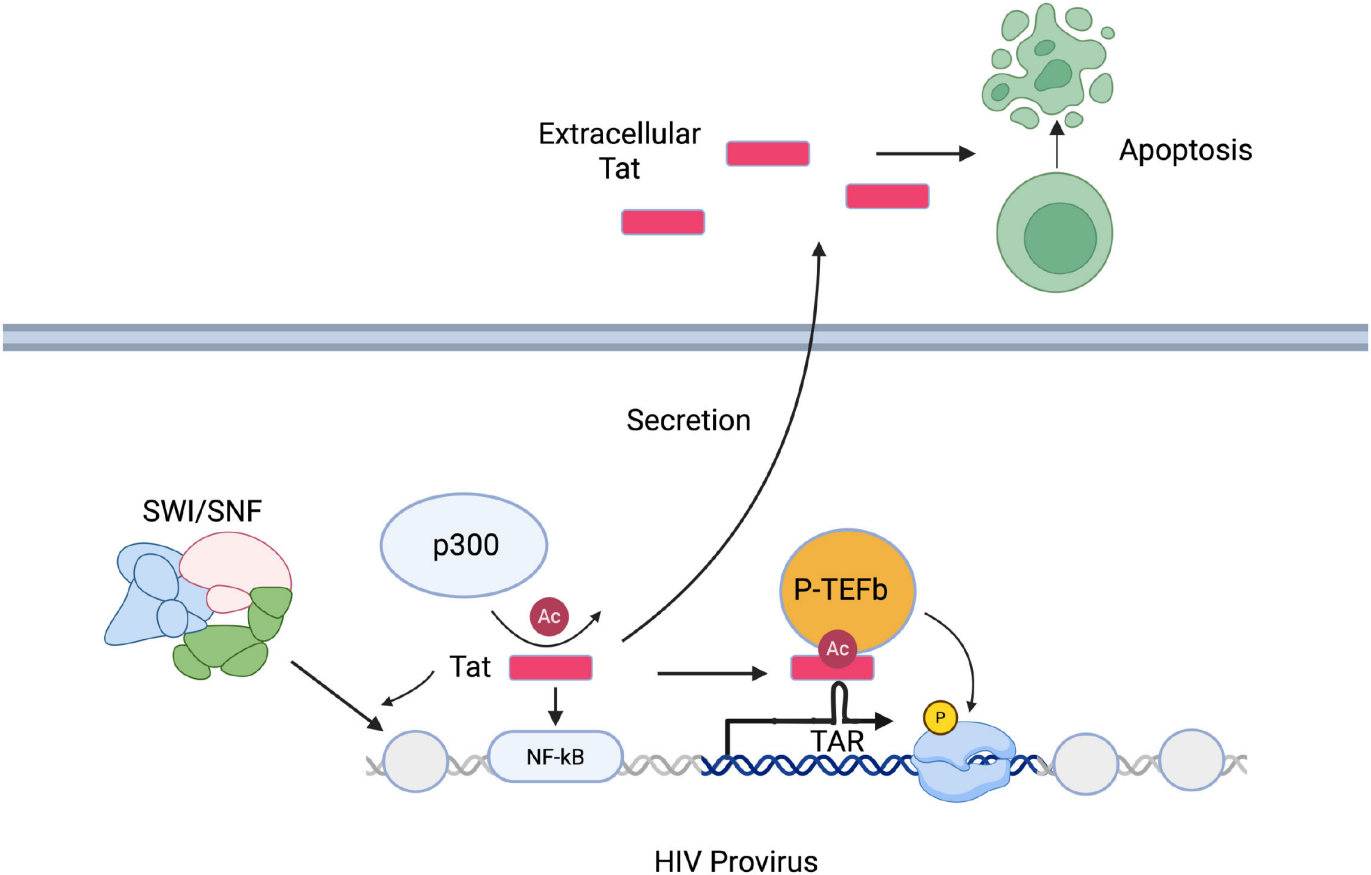
Roles of TAT in HIV expression.

In addition to its role in promoting the release of paused viral transcription complexes and HIV transcriptional elongation, Tat plays other important roles in HIV gene expression (**Figure 1**). Expression of Tat or delivery of Tat protein to cells has been shown to trigger rapid activation of NF-κB, a key HIV activating transcription factor (49). Tat also recruits acetyltransferases such as p300 and CREB-binding protein to the viral promoter (50, 51). Notably, Tat is acetylated at Lysine 28 by p300 and this enhances its activity by promoting interaction with Cyclin T1 (52). Additionally, Tat can induce nucleosome remodeling at the viral LTR by binding and recruiting members of the SWI/SNF complexes (53, 54). Blocks to any of these activities in infected cells could represent a pathway to establishment of a latent viral state.

The activity of Tat has also been shown to be inhibited in latently infected cells by members of the bromodomain containing protein family, BRD2 and BRD4. Current data suggests that BRD4 acts as a cellular competitor to Tat that sequesters P-TEFb via interaction with acetylated histones (55). Consistent with this hypothesis, bromodomain and external domain inhibitors (BETis) can promote the release of P-TEFb, thereby allowing its recruitment to the Tat/TAR complex and reactivation of HIV from latency (56–59). However, some data suggests that the ability of BETi compounds to reactivate latent HIV is independent of Tat, indicating that alternative mechanisms may exist (60). The BETi iBET151 has also recently been shown recently to potently synergize with the non-canonical NF-κB agonist AZD5582 with respect to reactivation of latent HIV (61).

Data from cell-based models of HIV latency indicate that Tat expression potently counteracts the establishment of latently infected cells, suggesting that lack of sufficient Tat expression in infected cells is likely a key step towards the formation of a pool of latently infected cells (62). Some data also suggests that variation in Tat sequences may contribute to the formation of the latent HIV reservoir. Specifically, Tat sequences recovered from PWH on ART were more likely to exhibit lower HIV transcription-enhancing activity in a cell-based assay than matched Tat sequences from actively infected cells taken before therapy (63). However, it remains unclear if this association is due to preferential archiving of low activity Tat variants prior to ART or due to selection of low expressing proviruses post therapy. It will also be important to fully evaluate how differences in Tat or TAR sequences across various clades of HIV affect the size and stability of the reservoir as well as its ability to be reactivated by latency reversing agents.

## 5 Expression of Tat during latency

HIV latency is not absolute, and ongoing viral gene expression can be detected within a subset of infected cells during ART. Many of these remaining viruses represent defective sequences that cannot reactivate or replicate but can nonetheless still generate some viral transcripts or protein. Residual Tat expression could thus potentially contribute to ongoing perturbation of the immune system in PWH. The 101 amino acid form of Tat is immunogenic and antibodies against Tat are detectable in PWH (64). Tat protein has also been shown to be cytotoxic and can trigger apoptosis (49, 65–68). Due to the presence of a leader peptide, Tat is secreted from infected cells and can be internalized by neighboring cells, allowing it to exert biological effects beyond the infected cells themselves (67). The mechanism of Tat secretion involves the basic domain of Tat (residues 48-57) binding to phosphatidylinositol-4,5-bisphosphate, leading to either direct translocation across the plasma membrane or formation of a transmembrane pore, although release in exosomes is another possible route of Tat release (69). Notably, PWH on ART exhibit elevated levels of immune activation and senescence, as well as higher rates of clinical comorbidities such as heart and kidney disease, despite effective suppression of viral replication (70–74). Since ART does not inhibit HIV expression, it is possible that ongoing Tat expression and secretion from both intact and defective proviruses during ART contributes to these perturbations and clinical comorbidities. As such, developing small molecules to inhibit Tat expression or activity could provide some clinical benefit.

Tat expression during ART may be particularly important to CNS pathogenesis in PWH. HIV invades the brain during untreated infection, and this typically occurs within days of initial infection, before the initiation of ART (75). Within the CNS, the primary target of HIV is microglial cells (76–80). While HIV or SIV infected microglia can be detected in brain tissue from PWH and from Rhesus Macaques on ART respectively, it is unclear if microglia become latently infected with similar frequency as seen in T cells. Although epigenetic modulators similarly disrupt latency in microglia (80), mechanisms of transcriptional latency and silencing in microglial has been little studied. CNS invasion by HIV is associated with increased levels of neurocognitive comorbidities, including a broad spectrum of disorders referred to as HIV-associated neurocognitive disorder (HAND (81);) HAND is observed in up to 50% of PWH, and persists in the presence of ART. The underlying molecular mechanisms that drive HAND are complex and poorly understood, but evidence indicates that the viral Tat protein plays an important role in HAND (82). Tat protein is secreted from chronically infected microglial cells, and PWH on ART exhibit detectable Tat protein in their CNS (83). Furthermore, as described above, Tat protein can be internalized by neighboring cells and promotes toxicity to neurons, microglial activation, and cytokine secretion (82). Notably, transgenic rodents which express Tat in brain tissue exhibit neurocognitive impairment that resembles HAND (84). Overall, these data point to Tat expression during ART from a CNS reservoir as a vital part of pathogenesis in the CNS.

## 6 Tat and latency reversal

To eliminate the latent HIV reservoir, several approaches have been investigated. The most studied is a two-step approach, first employing the induction of viral gene expression by latency reversing agents (LRAs), seeking to generate enough viral antigen to render the cell detectable (and killable) by effector immune mechanisms (85). The second step employs the induction or provision of antiviral effectors, via the administration of a vaccine, antibodies, a cellular therapy, or other novel anti-HIV immunotherapy approaches (86).

Thus far, several different classes of LRAs have been developed that target specific cellular pathways involved in HIV repression, including histone deacetylase inhibitors, bromodomain inhibitors, PKC agonists and non-canonical NF-κB agonists. Many of these compounds are effective at reactivating latent HIV proviruses in cell-based models, and some have also proven effective as LRAs *in vivo* in animal models of suppressed HIV or SIV infection (87, 88). HDACis such as Vorinostat and Romidepsin have also been shown to reactivate the viral reservoir in ART-suppressed PWH following clinical dosing (28, 89–91). Despite these encouraging results, no LRA has yet been shown to cause a significant decrease in the overall size of the reservoir *in vivo* (92). The reasons for this lack of potency for reservoir elimination are unclear but could be due to the reservoir being preferentially seeded in cells that are innately resistant to cell death pathways induced by HIV proteins (93). Additionally, work has shown that reactivation of the clinical reservoir, even by “potent” LRAs is typically inefficient, with only up to 10% of proviruses responding to a given round of stimulation (94). LRAs that target host cell pathways also lack specificity for HIV and target broadly conserved regulators of cellular transcription, leading to off-target effects on the exposed cells that could potentially promote toxicity or limit clinical usefulness (95). As such more broadly active and virus-specific approaches to latency reversal are needed.

Since Tat is a potent activator of viral gene expression that is specific to HIV, delivery or expression of Tat in latently infected cells could represent an ideal approach to improve clinical latency reversal. Despite this, delivery of Tat has received surprisingly little attention from researchers thus far. As a therapeutic tool, Tat presents several challenges. Tat is an intrinsically disordered protein and can thus be difficult to express, purify and formulate for clinical delivery (96). Also, the cytotoxic characteristics of Tat should trigger caution for clinical use. Nevertheless, some studies have begun to examine the potential of Tat as an LRA approach. An important limitation of most of these studies so far is that Tat delivery has largely been tested in cell lines, primary cell models and, in some cases *ex vivo* blood cells from PWH on ART. It will be vitally important to expand testing of Tat delivery to animal models and to cells from peripheral tissue such as the gastrointestinal tract, where the majority of HIV infected cells reside (97, 98).

The earliest efforts to deliver Tat to latently infected cells involved delivery of intact Tat protein. Lin et al. demonstrated that delivery of an exogenous recombinant Tat-GST fusion protein could transactivate latent proviruses in cells from PWH on ART (99), while Donahue et al. (62) demonstrated that exposure to recombinant Tat protein prevented the emergence of a latently infected population of cells in a Jurkat cell line. This study also demonstrated that a variant of Tat with enhanced transactivation activity (T32N) exhibited higher activity against latency. Since full-length Tat protein is both cytotoxic and immunogenic, subsequent studies have focused on truncated forms of Tat that lack part of the immunogenic C terminal domain (100). Previous structure-function analysis of Tat has showed that residues 87–101 are immunogenic and not required for trans-activating ability. Another study (101) confirmed that delivery of recombinant Tat protein reverses latency, and that an 86 amino acid truncated version containing five point mutations (T5R4) was almost as potent as full length Tat, but exhibited less immunogenicity and cytotoxicity.

In addition to the demonstrated potency of recombinant Tat protein, superinfection of latently infected cells with HIV reactivated latent proviruses in a Tat-dependent manner (102).

Due to the success of lipid nanoparticle (LNP) based mRNA delivery for vaccination, delivery of Tat mRNA has recently been tested as a novel approach for latency reversal. Van Gulck et al. used LNPs containing a truncated Tat mRNA to demonstrate that these particles can reactivate both viral RNA expression and p24 protein to a level similar to the potent T cell activator PMA/ionomycin (103). The authors were also able to use this approach to activate viral RNA in cells from PWH in order to enhance detection of infected cells through single cell transcriptomic profiling, and thereby identify a specific signature of gene expression associated with HIV infection in PWH on ART. More recently, Pardons et al. have further refined this approach by demonstrating that delivery of an mRNA encoding the N terminal 66 amino acids of Tat (T66), or recombinant T66 protein, was as potent as full-length Tat at promoting latency reversal. A combination of Tat-mRNA LNPs and the class 1 HDACi Panobinostat significantly outperformed PMA/Ionomycin, and Tat delivery caused minimal alterations to the transcriptome of exposed cells (104).

In new work (105), we have also recently investigated the ability of Tat mRNA LNPs to combine with other benchmark LRAs. Specifically, we observed that Tat mRNA was able to reactivate HIV synergistically with IAPi, HDACi, and BETi LRAs, indicating a fairly broad potential for Tat/LRA synergy. Notably, this activity dependent on the HIV TAR loop, indicating that P-TEFb recruitment and transcriptional elongation represents the primary mechanism by which Tat mRNA promotes HIV reactivation. Consistent with this hypothesis, Tat mRNA promoted the expression of elongated, multiply-spliced, and polyadenylated HIV transcripts but not paused TAR RNA species. Tat mRNA was also observed to induce detectable viral p24 protein in PWH-derived samples at 48h post exposure, suggesting that this approach is sufficient to induce viral antigen.

Despite these promising results, it is important to consider challenges relating to delivery of Tat mRNA as a therapeutic strategy for PWH. LNPs have significant obstacles for systemic delivery – intravenously injected LNPs typically undergo rapid coating by the serum protein ApoE followed by LDL receptor-dependent uptake in the liver, and thus poorly penetrate important tissues such as the brain (106). Improved LNP formulations to avoid liver targeting or to promote targeting to specific cell types by functionalization with antibodies may be useful for mitigating these issues (107). Delivery of mRNA also requires translation of the Tat transcript, and translation could be a barrier to high Tat expression in reservoir cells due the reservoir being present in quiescent resting memory cells with low metabolic activity. An additional concern for Tat delivery is that Tat protein expressed in tissues such as the brain could be secreted and exacerbate HAND pathology via bystander toxicity. It will thus be crucial to fully evaluate the potency and toxicity of Tat-based therapeutics for HIV infection in animal models before clinical deployment. It will also be important to determine how to best integrate Tat delivery with immune clearance tools to achieve effective reduction of the HIV reservoir.

## 7 Inhibiting Tat to silence the HIV reservoir

In recent years, a new approach to targeting the HIV reservoir has emerged, in which inhibitors of viral transcription are used to induce a prolonged or permanent state of viral repression, referred to as “block and lock” (108). The underlying hypothesis behind this approach is that ongoing residual transcription of the provirus in latently infected cells helps to maintain a transcriptionally poised state, and that temporary inhibition of this residual viral transcription in latently infected cells could promote the formation of long-lived repressive epigenetic changes at HIV. Over the past few years, several small molecules that target Tat or the TAR RNA have been identified in biochemical screens and some have shown promise in inhibiting HIV expression and/or virus replication. The nine-residue peptide/peptoid CGP64222 induces a conformation change in TAR that inhibits HIV expression (109). By contrast, the Stilbene derivate CGA137053 was shown to directly bind to Tat protein and inhibit HIV replication (110). Additionally, some compounds have been found that block Tat-dependent HIV transcription indirectly - a high throughput screen identified the GSK3 inhibitor 6BIO as a potent inhibitor of Tat-dependent expression *in vitro* and in cell-based assays (111). The coumarin derivative BPRHIV001has also been shown to inhibit Tat-dependent expression by affecting stability of p300, a cellular lysine acetyl transferase that enhances Tat activity by acetylation of Tat on lysine 28 (112). Despite this progress, few of these compounds have been directly tested in primary cell latency models or in cells from PWH on ART for their ability to block HIV reactivation from latency. An important recent development in the ‘Block and Lock’ approach came with the identification of didehydrocotistatin A (dCA) as a potent and selective inhibitor of Tat that binds to the basic unstructured region of Tat protein an inhibits its transcription activating function with nanomolar affinity (113, 114). Exposure of latently infected cells to dCA inhibits viral reactivation and particle release, even after dCA removal, indicating a long-lasting activity against latent proviral viral expression. Notably, dCA dosing of ART suppressed HIV infected humanized mice delays viral rebound after ART interruption (115).

## 8 Concluding remarks

Despite its central importance to HIV expression, Tat remains understudied as a target to promote latency reversal or to inhibit viral reactivation. Although several compounds that inhibit Tat have been identified, many present challenges with synthesis or bioavailability, and few have been tested in relevant latency model systems for their ability to affect viral reactivation. Tat protein levels are likely a key limiting factor for the overall potency of latency reversal approaches and for activating Tat-dependent transcription in latently infected cells. Other key barriers to a clinically meaningful reversal of HIV latency, including the epigenetic state of the provirus, and the overall transcriptional state of the cell, are approachable by current LRAs, and new molecules entering the clinic (116). The delivery of Tat protein or mRNA holds significant promise as a way to overcome a key remaining barrier and promote HIV expression in a virus-specific manner. This promise, however, is tempered by concerns about delivery and off-target toxicity of Tat. Further testing of Tat in combination with existing LRA approaches could lead to clinically significant latency reversal. Achieving this first key step would allow for rational testing of immunotherapies towards clearance of persistent HIV infection.
